# Supplementary material for: Status, rivalry and admiration-seeking in narcissism and depression: A behavioral study
Source: PLoS One. 2020 Dec 3;15(12):e0243588. doi: 10.1371/journal.pone.0243588 (PMC7714187; doi:10.1371/journal.pone.0243588)
Supplement: S1 Appendix — (PDF) [file pone.0243588.s001.pdf]

S1 Appendix

	Sample 1	Sample 2		
		**TOTAL**	healthy	depressed
n	**70**	**85**	25	60
Age (mean (sd))	21.46 (3.34)	62.62 (7.98)	65.04 (8.92)	61.62 (7.39)
Female sex (%)	55 (78.6)	51 (60.0)	16 (64.0)	35 (58.3)
Race (%)				
Caucasian	14 (20.9)	68 (80.0)	23 (92.0)	45 (75.0)
Asian	39 (58.2)	2 (2.4)	0 (-)	2 (3.3)
African-American/African-Canadian	1 (1.5)	14 (16.5)	2 (10.0)	12 (20.0)
Other	13 (19.4)	1 (1.2)	0 (-)	1 (1.7)
Household income in USD (mean (sd))	----	43,148.81 (31,212.52)	64,020.00 (28399.12)	34,305.08 (28161.94)
Household income in CAD (%)				
< 25,000	6 (9.0)	----	----	----
25,000 - 49,999	10 (14.9)	----	----	----
50,000 - 74,999	13 (19.4)	----	----	----
75,000 - 99,999	15 (22.4)	----	----	----
100,000 - 149,999	12 (17.9)	----	----	----
≥ 150,000	11 (16.4)	----	----	----
Game experience (mean (sd))	1.79 (1.30)	2.55 (1.51)	2.68 (1.44)	2.50 (1.55)
DASS-21 depression subscale (mean (sd))	9.51 (8.86)	----	----	----
HRSD (mean (sd))	----	12.14 (9.03)	1.80 (1.96)	16.45 (7.09)
Depression severity percentile based on general population norms* (mean (sd))	59.46 (24.93)	62.51 (34.11)	17.60 (16.82)	81.22 (18.18)
FFNI total score (mean (sd))	153.21 (28.16)	134.36 (25.49)	127.68 (17.77)	137.34 (27.90)

Table A. Characteristics of Sample 1 and 2. *See Methods – Statistical analysis for a description of how depression percentile norms were obtained.

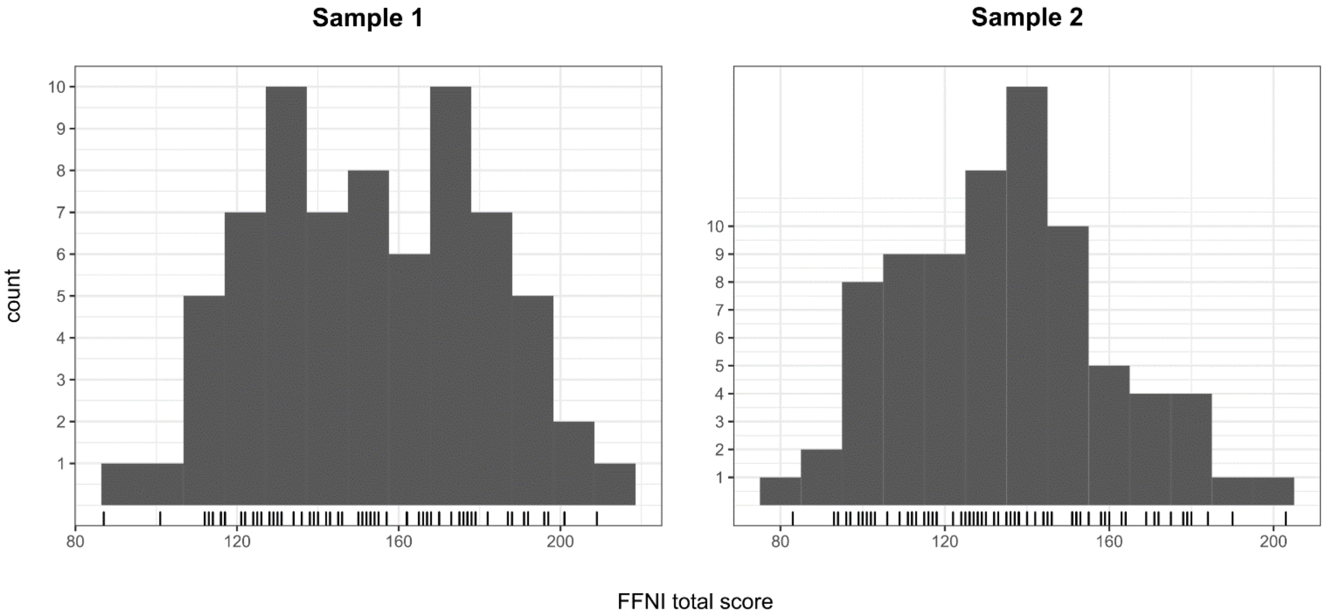

Fig A. Distribution of narcissistic scores in the two samples. FFNI, Five-Factor Narcissism Inventory (scoring range: 60-300).

Measured construct	Scale	Sample 1	Sample 2
		Cronbach's α	
Trait dominance	IPIP-DS	Scale not collected	.84
Narcissism	FFNI	.92	.90
	Agentic Extraversion	.88	.85
	Antagonism	.92	.89
	Narcissistic Neuroticism	.86	.90
	BPNI	.92	.95
Depression	DASS-21 depression	.88	Scale not collected
	HRSD	Scale not collected	.89

Table B. Reliability of psychometric measures used in the study. Legend: IPIP-DS, International Personality Item Pool Dominance Subscale; FFNI, Five-Factor Narcissism Inventory; BPNI, Brief Pathological Narcissism Inventory; HRSD, Hamilton Rating Scale for Depression.

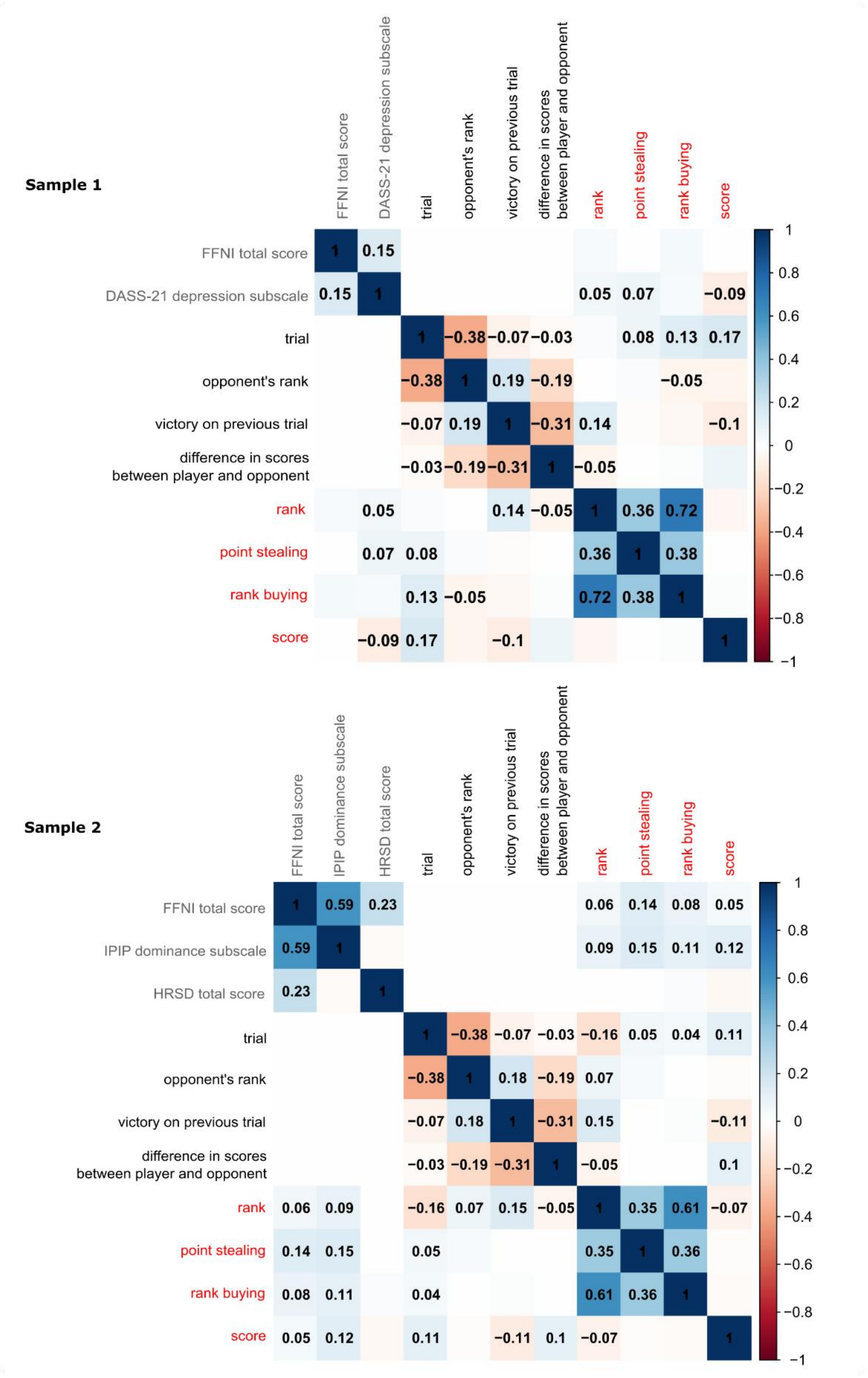

Fig B. Spearman correlations between psychometric and task-related variables. In grey: psychometric variables, in black: preset design variables (uniform for all participants), in red: behavior-dependent variables. Numbers indicate coefficients of significant correlations ($p < .05$). Legend: FFNI, Five-Factor Narcissism Inventory; HRSD, Hamilton Rating Scale for Depression; *, $p < .05$; **, $p < .01$; ***, $p < .001$.

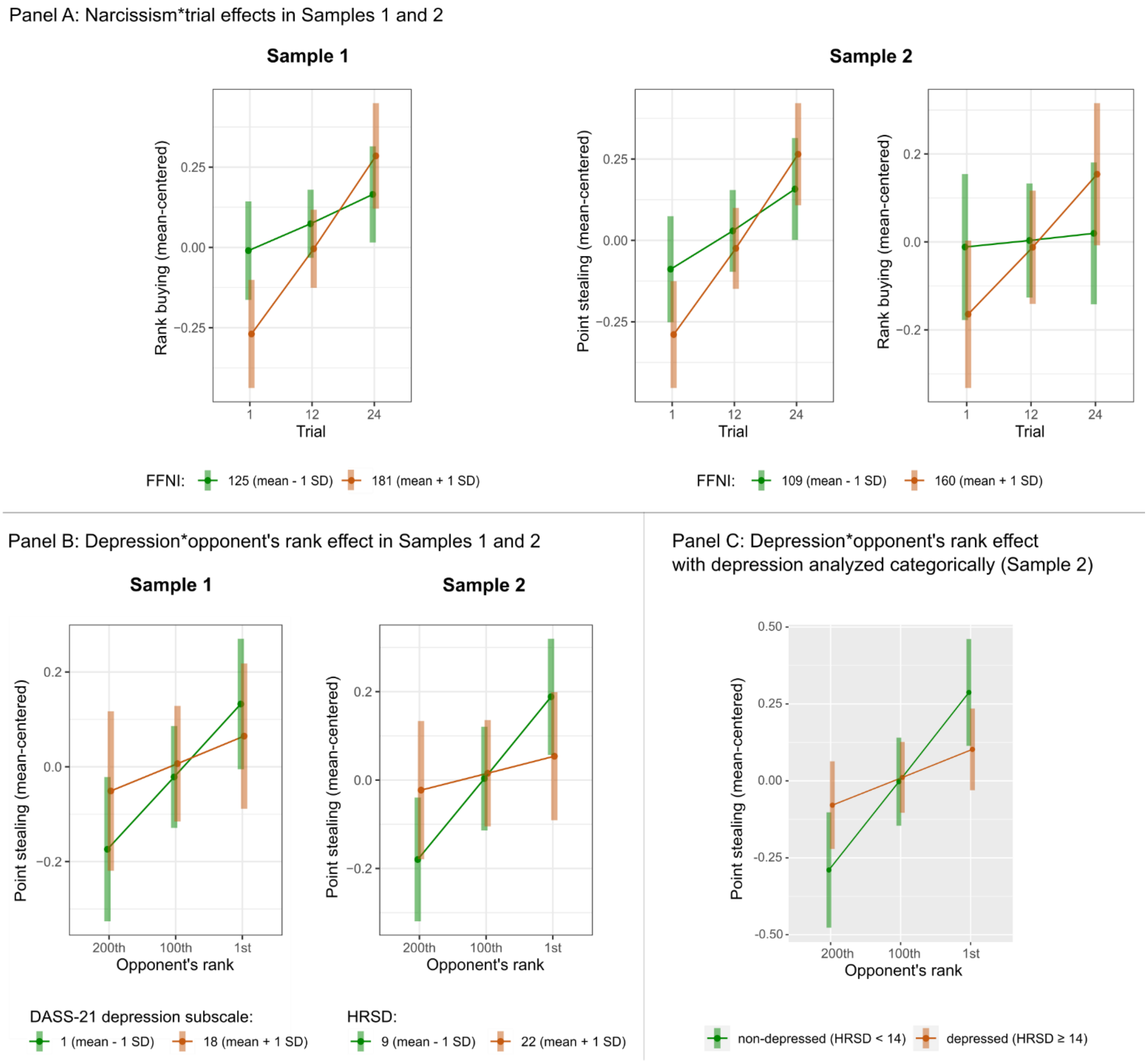

Fig C. Panel A: *Narcissism*trial* effects in the individual samples, indicating a steeper increase of point stealing and rank buying over time in more narcissistic participants. The *narcissism*trial* effect predicting point stealing was not significant in Sample 1 (not shown), the other effects are consistent in their pattern and direction. **Panel B: *Depression*opponent's rank* effect predicting point stealing in the individual samples,** indicating that more depressed individuals tended to steal points indiscriminately of their opponent's rank. The effect had a consistent pattern across samples but did not survive the inclusion of covariates in Sample 1 (plots shown in figure were generated from models including covariates). **Panel C: The *depression*opponent's rank* effect predicting point stealing remained significant when depression was analyzed categorically in Sample 2.** Points indicate estimates and vertical bars 95% confidence intervals. Legend: FFNI, Five-Factor Narcissism Inventory; HRSD, Hamilton Rating Scale for Depression.

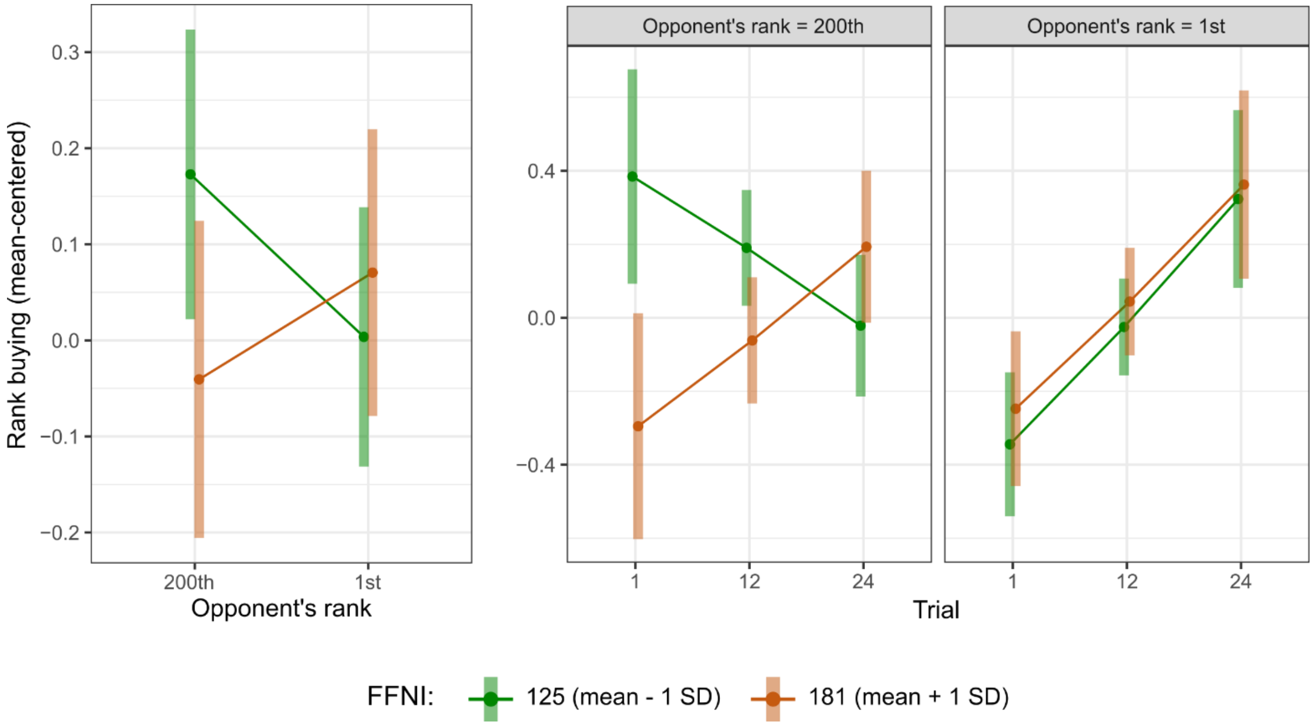

Fig D. *Narcissism*opponent's rank* (left) and *narcissism*opponent's rank*trial* effects (right) predicting rank buying in Sample 1, indicating that although more narcissistic participants bought rank preferentially against high-ranked opponents, they also increased rank buying against low-ranked opponents over time. Less narcissistic individuals had an opposite tendencies, buying rank preferentially against low-ranked opponents, but decreasing rank buying against them with time. These effects were significant neither in Sample 2 nor in the pooled analysis, and thus were not retained as main findings. Points indicate estimates and vertical bars 95% confidence intervals. Legend: FFNI, Five-Factor Narcissism Inventory.

Sensitivity analyses

(a) Long-string responding

	Sample 1 (N = 70)	Sample 2 (N = 85)
Point stealing	15/70 (21%)	18/85 (21%)
Rank buying	13/70 (19%)	15/85 (18%)
Both measures	9/70 (13%)	10/85 (12%)

Table C. The proportion of long-string responders per sample and outcome variable. Rates were comparable between the two samples and did not exceed 13% of participants in total.

	Sample 1			Sample 2		
	**Long-string responders**	**Other participants**	***p***	**Long-string responders**	**Other participants**	***p***
n	9	61	----	10	75	----
Age (mean (sd))	20.78 (1.56)	21.56 (3.53)	0.518	63.40 (8.30)	62.52 (7.98)	0.745
Female sex (%)	8 (88.9)	47 (77.0)	0.709	4 (40.0)	47 (62.7)	0.303
Race (%)			0.974			**0.021**
Caucasian	2 (22.2)	12 (20.7)		9 (90.0)	59 (78.7)	
Asian	5 (55.6)	34 (58.6)		0 (0.0)	2 (2.7)	
African-American/-Canadian	0 (0.0)	1 (1.7)		0 (0.0)	14 (18.7)	
Other	2 (22.2)	11 (19.0)		1 (10.0)	0 (0.0)	
Household income in USD (mean (sd))	----	----	----	50800.00 (34892.06)	42114.86 (30797.34)	0.412
Household income in CAD (%)			0.925	----	----	----
< 25,000	0 (0.0)	6 (10.3)				
25,000 - 49,999	1 (11.1)	9 (15.5)				
50,000 - 74,999	2 (22.2)	11 (19.0)				
75,000 - 99,999	2 (22.2)	13 (22.4)				
100,000 - 149,999	2 (22.2)	10 (17.2)				
≥ 150,000	2 (22.2)	9 (15.5)				
Game experience (mean (sd))	2.11 (1.69)	1.74 (1.24)	0.424	2.70 (1.83)	2.53 (1.47)	0.745
DASS-21 depression subscale (mean (sd))	4.44 (5.17)	4.80 (4.35)	0.822	----	----	----
HRSD (mean (sd))	----	----	----	12.67 (7.70)	16.02 (6.09)	0.149
FFNI total score (mean (sd))	155.44 (30.70)	152.89 (28.03)	0.801	131.56 (32.11)	134.71 (24.80)	0.729

Table D. Long-string responders compared to other participants on demographic and psychometric measures. Long-string responders did not differ significantly from other participants, with the sole exception of race in Sample 2 (in bold).

**Main findings retested after exclusion of long-string responders**	**Reduced Sample 1 (N = 61)**	**Reduced Sample 2 (N = 75)**	**Pooled (N = 136)**
	Coefficient (standard error)		
(i) Reaction to defeat			
Point stealing			
Point stealing increases over time	**.151 (.031)*** **	**.131 (.042)*** **	**.144 (.020)*** **
Point stealing increases more over time in participants with higher levels of narcissism	.034 (.026)	**.054 (.023)* **	**.052 (.017)** **
Rank buying			
Rank buying increases over time	**.120 (.029)*** **	**.051 (.026)* **	**.084 (.019)*** **
Rank buying increases more over time in participants with higher levels of narcissism	**.069 (.028)* **	**.049 (.023)* **	**.061 (.017)*** **
(ii) Level of social comparisons			
Point stealing			
Point stealing increases against high-ranked opponents	**.079 (.028)** **	**.108 (.024)*** **	**.097 (.018)*** **
Point stealing increases more against high-ranked opponents after having performed well on the arcade game	**.053 (.024)* **	**.059 (.022)** **	**.038 (.016)* **
Point stealing does **not** increase against high-ranked opponents in highly depressed participants	-.040 (.028)	**-.073 (.021)*** **	**.067 (.016)*** **
Rank buying			
Rank buying increases more over time against high-ranked opponents	**.067 (.024)** **	.008 (.022)	**.034 (.017)* **

Table E. Sensitivity analysis of main findings after exclusion of participants with long-string responses on both point stealing and rank buying. Significant effects are in bold. All main findings remained significant in the reduced samples. Legend: *, p <.05; **, p <.01; ***, p<.001.

(b) Effects of sex

**Main findings retested in models covarying for *sex*trial*, *sex*outcome* and *sex*opponent's rank***	**Sample 1 (N = 70)**	**Sample 2 (N = 85)**	**Pooled (N = 155)**
	Coefficient (standard error)		
(i) Reaction to defeat			
Point stealing			
Point stealing increases over time	**.126 (.029)*****	**.118 (.029)*****	**.127 (.020)*****
Point stealing increases more over time in participants with higher levels of narcissism	.025 (.022)	**.054 (.021)***	**.044 (.015)****
Rank buying			
Rank buying increases over time	**.125 (.028)*****	.047 (.029)	**.089 (.020)*****
Rank buying increases more over time in participants with higher levels of narcissism	**.060 (.024)***	**.053 (.022)***	**.057 (.015)*****
(ii) Level of social comparisons			
Point stealing			
Point stealing increases against high-ranked opponents	**.078 (.027)****	**.089 (.027)****	**.080 (.019)*****
Point stealing increases more against high-ranked opponents after having performed well on the arcade game	**.056 (.023)***	**.049 (.019)***	**.031 (.014)***
Point stealing does **not** increase against high-ranked opponents in highly depressed participants	-.032 (.024)	**-.064 (0.19)*****	**-.057 (.014)*****
Rank buying			
Rank buying increases more over time against high-ranked opponents	**.059 (.021)****	.008 (.020)	**.030 (.014)***

Table F. Sensitivity analysis of main findings covarying for *sex*trial*, *sex*outcome* and *sex*opponent's rank*. Significant effects are in bold. All effects were robust to the additional covariates, with the exception of the main effect of trial predicting rank buying in Sample 2 (highlighted in yellow), which nevertheless maintained a similar effect magnitude as in the principal model. Coefficients were computed with female sex as the reference group. Legend: *, p <.05; **, p <.01; ***, p<.001.

Effects tested	Sample 1	Sample 2	Pooled
	Coefficients (standard error)		
Point stealing			
Male sex***trial**	.047 (.054)	-.019 (.041)	-.010 (.032)
Male sex***narcissism*trial**	-.005 (.053)	-.038 (.043)	-.007 (.033)
Male sex***opponent's rank**	-.056 (.055)	.027 (.039)	.023 (.030)
Male sex***opponent's rank*previous score**	-.057 (.049)	.012 (.039)	-.037 (.028)
Male sex***depression*opponent's rank**	.001 (.069)	.046 (.040)	.049 (.030)
Rank buying			
Male sex***trial**	**-.138 (.051)****	-.034 (.042)	**-.083 (.032)****
Male sex***narcissism*trial**	-.095 (.051)	.003 (.044)	-.049 (.033)
Male sex***opponent's rank*trial**	-.070 (.050)	**-.088 (.040)***	**-.087 (.031)****

Table G. Main findings tested for moderation by sex. Significant effects are in bold. Sex interacted with *trial* and *opponent's rank*trial* effects predicting rank buying. Sex did not moderate any of our main findings involving narcissism or depression. Legend: *, p <.05; **, p <.01.

Exploratory analyses

(b) Effects of trait dominance

**Significant effects**	**Sample 2 (N = 85)**
	Coefficient (standard error)
(i) Reaction to defeat	
**Point stealing** increases more over time in participants with higher levels of trait dominance; *trial*trait dominance* effect.	**.062 (.023)****
**Rank buying** increases more over time in participants with higher levels of trait dominance; *trial*trait dominance* effect.	**.051 (.021)***
(ii) Level of social comparisons	
**Point stealing** increases against high-ranked opponents in participants with higher levels of trait dominance; *opponent's rank*trait dominance* effect.	**.071 (.021)*****

Table H. Significant interaction effects with trait dominance in Sample 2, where this measure was available**.** Legend: *, p <.05; **, p <.01.

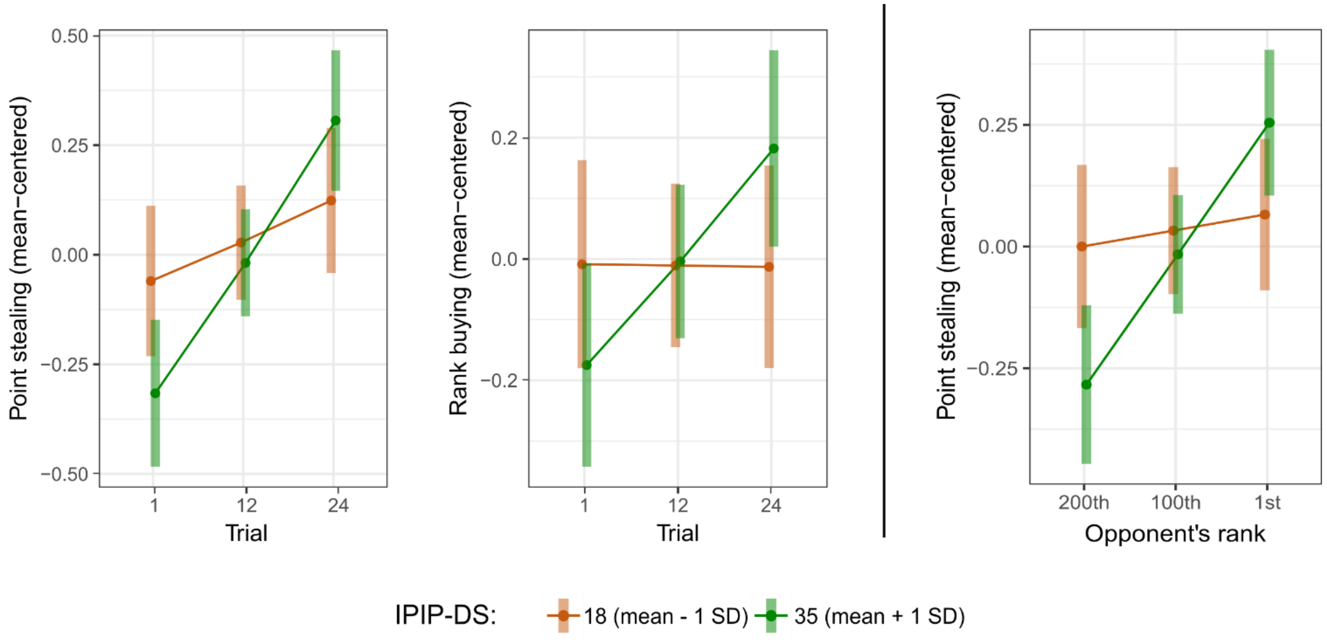

Fig E. Left panel: Significant *trait dominance*trial* interactions predicting point stealing and rank buying in Sample 2, where the IPIP-DS was collected. Similarly to narcissism, trait dominance increased point stealing and rank buying throughout time (number of trials). **Right panel: Significant *trait dominance*opponent's rank* interaction predicting point stealing in Sample 2**, indicating that individuals high on trait dominance preferentially stole points from high-ranked opponents. Points are estimates from the corresponding regression model at the indicated values; vertical bars represent 95% confidence intervals. Legend: FFNI, Five-Factor Narcissism Inventory.

(c) Performance on the snake arcade game

As in the main text, we primarily report findings in terms of the pooled analysis but detail discrepancies between samples (Fig F).

Trials had a significant main effect (χ^2_1 = 104.95, p < .001). Younger age (χ^2_1 = 133.71, p < .001), male sex (χ^2_1 = 39.48, p < .001), having lost on the previous trial (χ^2_1 = 65.76, p < .001), being pitted against a high-ranked opponent (χ^2_1 = 8.89, p = .003), game experience (χ^2_1 = 10.78, p = .001) and being Caucasian vs. Asian (χ^2_3 = 8.86, p = .031) also predicted better performance. In Sample 1, opponents' rank did not influence scores significantly, whereas in Sample 2, game experience and race did not show significant main effects.

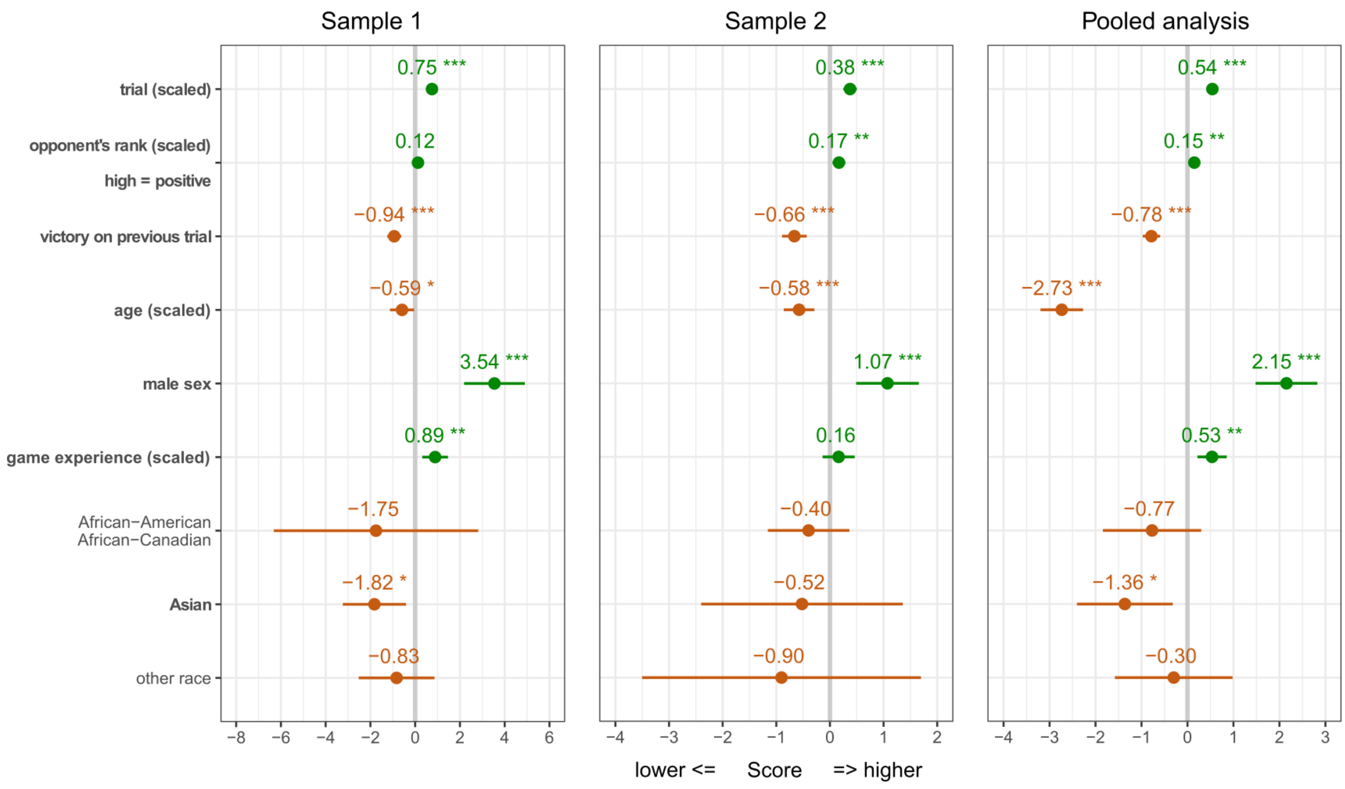

Fig F. Linear regression models predicting scores (task performance) in the two samples and the pooled analysis. Levels of *race* are compared to *Caucasian*. Effects significant in the pooled analysis are in bold. Points and numbers indicate estimates of fixed effects (negative estimates are displayed in orange); horizontal bars represent standard errors. Legend: *, p <.05; **, p <.01; ***, p <.001.

In the pooled analysis, sample was added to all independent variables as an interaction term, after removing age from the model to avoid multicollinearity. Here, significant *sample*trial* (χ^2_1 = 12.44, p < .001), *sample*sex* (χ^2_1 = 11.34, p < .001) and *sample*game experience* (χ^2_1 = 4.22, p < .040) effects

emerged, indicating that participants' scores in Sample 1 were overall higher and more steeply improving than in Sample 2, and that the difference in performance was larger in Sample 1 for men compared to women and for more experienced participants compared to less experienced ones (Fig G, Panel A).

Last, *narcissism*trial* and *depression*trial effects* were tested in the initial model (Table I and Fig G, Panel B). A significant *narcissism*trial* effects (χ^2_1 = 7.65, *p* = .006) suggested a higher task engagement in participants in more narcissistic participants. With respect to narcissistic subscales (Supplemental Table I), the effect was driven by agentic extraversion (χ^2_1 = 8.98, p = .003) and to a lesser extent by antagonism (χ^2_1 = 4.12, p = .042). Antagonism nevertheless predicted lower scores overall (χ^2_1 = 4.37, *p* = .037), similarly to BPNI total scores (χ^2_1 = 7.61, *p* = .006). Depression also had a negative main effect (χ^2_1 = 4.53, *p* = .033), but did not influence performance over time (χ^2_1 = .17, *p* = .677).

**Outcome variable: score**	**Main effect**	**Psychometric measure*trial**
	Coefficient (standard error)	
FFNI TOTAL SCORE	-.331 (.175).	**.135 (.049)****
FFNI Agentic extraversion	-.002 (.170)	**.146 (.049)****
FFNI Antagonism	**-.357 (.171)***	**.098 (.048)***
FFNI Narcissistic neuroticism	-.119 (.166)	-.008 (.049)
BPNI TOTAL SCORE	**-.487 (.176)****	.086 (.050).
DEPRESSION	**-.331 (.156)***	-.020 (.048)

Table I. Psychometric measures predicting task performance in the pooled analysis. Significant effects are in bold. Left column: depression, BPNI total scores and, FFNI antagonism predicted a poorer overall performance (significant main effect). Right column: FFNI total scores, agentic extraversion and antagonism predicted a greater improvement in task performance over time (positive interaction with trial). Legend: FFNI, Five-Factor Narcissism Inventory; BPNI, Brief Pathological Narcissism Inventory; *, p <.05; **, p <.01.

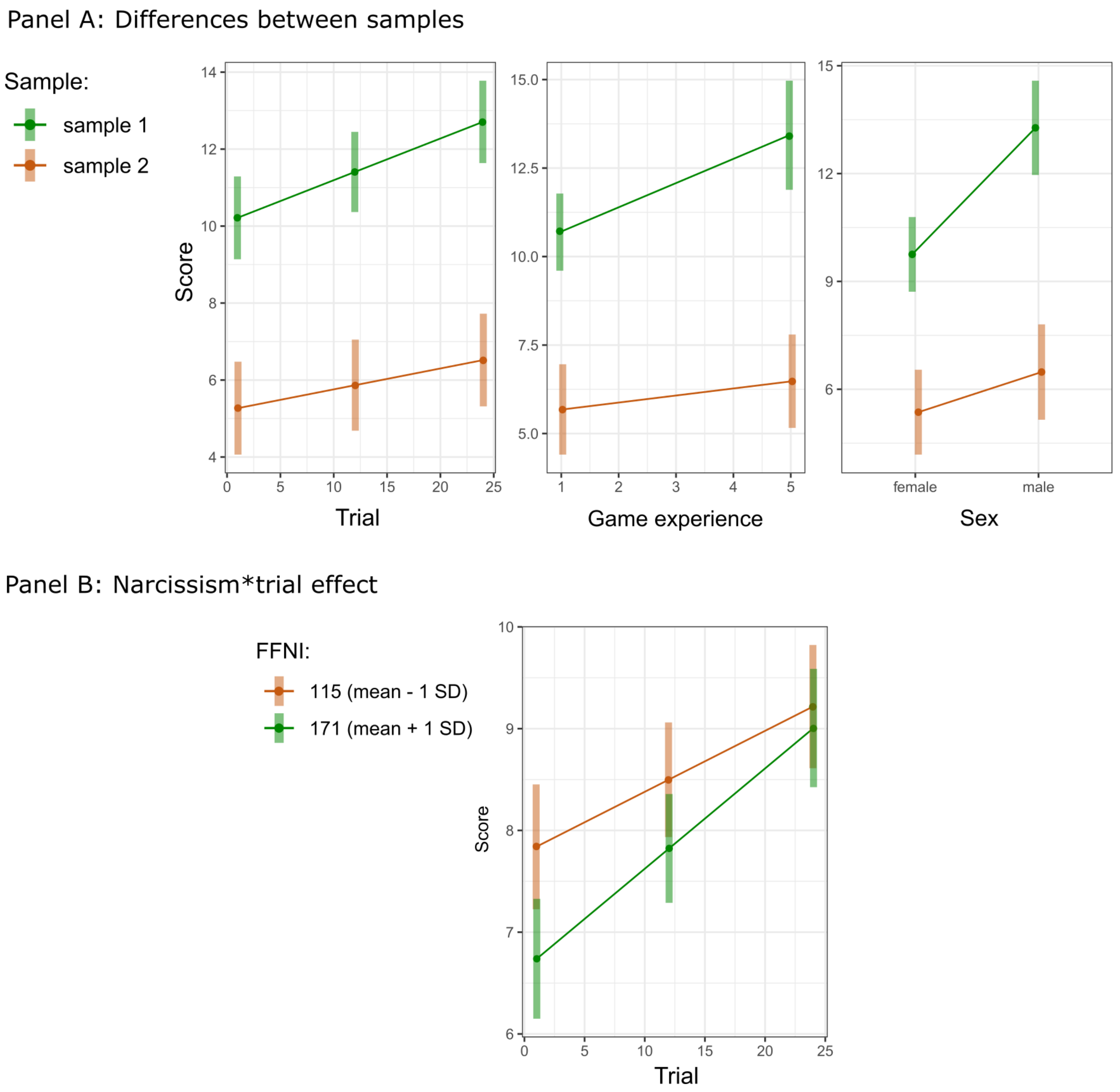

Fig G. Panel A: Differences between samples predicting task performance (score) in the pooled analysis. The direction of effects were similar but differences in performance with respect to trial (time on task), game experience and sex were larger in Sample 1. **Panel B: *Narcissism*trial* interaction predicting task performance.** Narcissism further enhanced the improvement rate of task performance. Points indicate estimates and vertical bars 95% confidence intervals.

(d) Correlations with participants' self-reported motivations and impressions

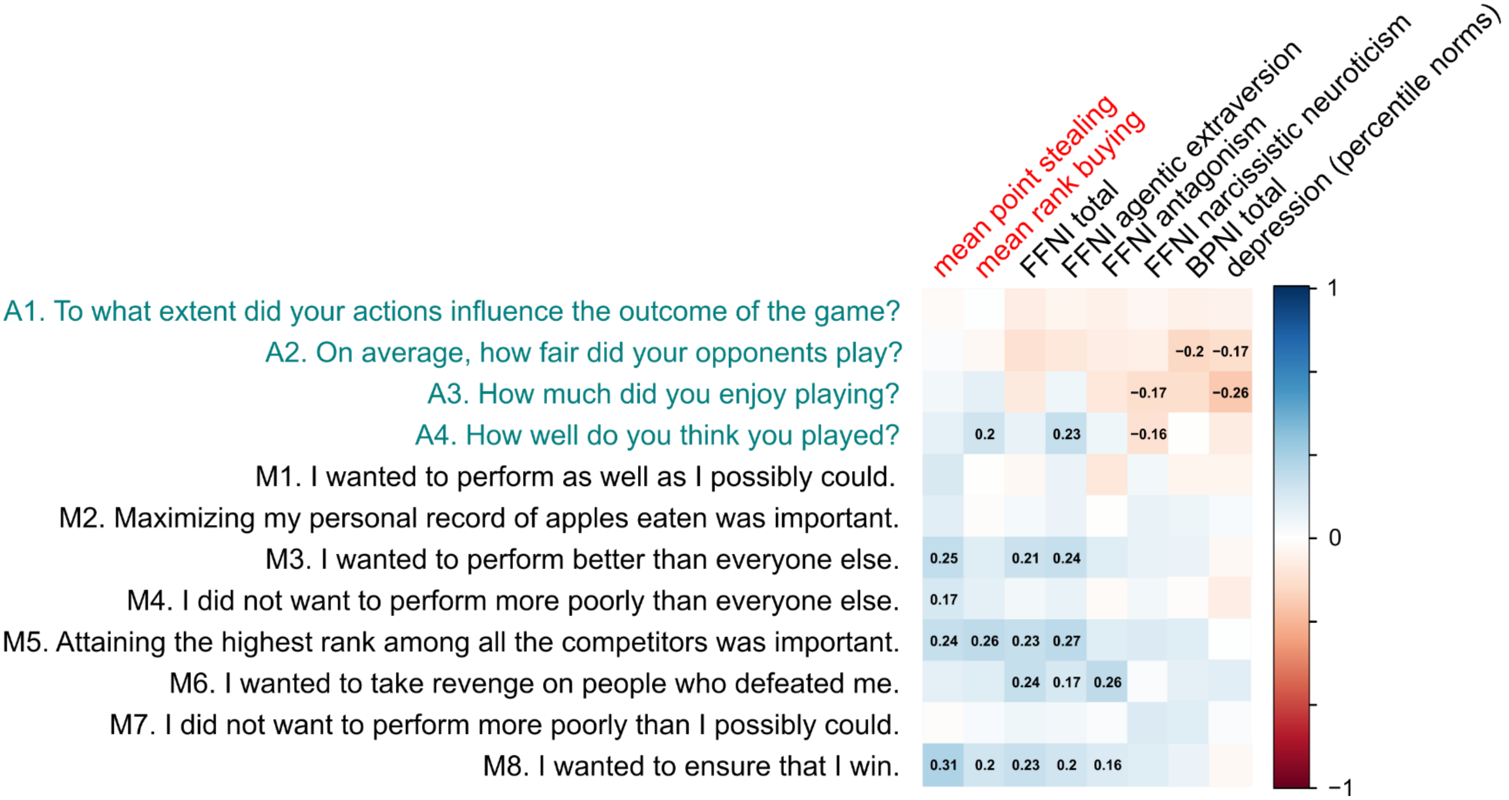

Fig H. Spearman correlations of mean point stealing and rank buying and psychometric measures with participants' self-reported feedback in the pooled analysis. Numbers indicate coefficients of significant correlations (p < .05). Questions A1 to A4 (in blue) investigated participants' impressions about the task and were answered on an analog scale from 0 (*not at all*) to 10 (*absolutely*). Questions M1 to M8 (in black) explored participants' motivations throughout the competition on a five-point Likert scale going from 1 (*strongly disagree*) to 5 (*strongly agree*). Participants encountered the eight M questions and then the four A questions at the very end of the task, as these questions were built in the tournament's interface. See main text for a description of findings. Legend: FFNI, Five-Factor Narcissism Inventory; BPNI, Brief Pathological Narcissism Inventory.
